# I’m Not Creative in That Way: Older Adults’ Differential Experiences of an Arts-Based Intervention

**DOI:** 10.1093/geroni/igaa057.3058

**Published:** 2020-12-16

**Authors:** Colette Brown, Andrea Chirino, Cristina Cortez, Cassandra Gearhart

**Affiliations:** 1 University of California, Irvine, Irvine, California, United States; 2 Chapman University, Orange, California, United States; 3 University of Southern California, Los Angeles, California, United States; 4 University of Texas at Austin, Austin, Texas, United States

## Abstract

A recent 12-week intervention study revealed that making conceptual art is linked to improved cognitive health among community-dwelling older adults (Brown et al., 2020). Unknown, however, is whether the intervention experience differed for participants who exhibited more versus less improvement. This pilot study examined 163 excerpts from semi-structured interviews with cognitively normal, older adult participants (N = 11, Mean age = 72.82). Using thematic analysis and data displays on Dedoose, key themes were distilled regarding intervention acceptability. Participants exhibiting less cognitive improvement more often mentioned personally connecting to topics of dementia and aging through art, but more often mentioned scheduling conflicts. Conversely, participants exhibiting greater cognitive improvement more often mentioned experiencing intellectual enrichment, but feeling insecure about their art capabilities. Novel art activities may be personally meaningful and cognitively stimulating for some participants, but emotionally frustrating for others. Future work should explore ways to optimize arts-based interventions for older participants.

